# Occurrence of 3-monochloropropane-1,2-diol and glycidyl esters in artisanal vegetable edible oils

**DOI:** 10.1016/j.heliyon.2024.e34680

**Published:** 2024-07-16

**Authors:** Daniel Sitsofe Yabani, Isaac Williams Ofosu, Gloria Mathanda Ankar-Brewoo, Herman Erick Lutterodt

**Affiliations:** a New Products Development Unit, Cocoa Research Institute of Ghana, New Tafo-Akim, Ghana; b Food Systems Chemistry, Toxicology, and Risks Studies, Department of Food Science and Technology, College of Science, Kwame Nkrumah University of Science and Technology (KNUST), Kumasi, Ghana

**Keywords:** 3-Monochloropropane-1,2-diol, Glycidyl fatty acid esters, Glycidol, Artisanal oils, GC-MS

## Abstract

The safety of vegetable oils has come under intense scrutiny ever since the International Agency for Research on Cancer issued an alert on the carcinogenic properties of 3-monochloropropane-1,2-diol fatty acid esters (3-MCPDE) and glycidyl esters (GE). In this study, a total of 114 samples of artisanal palm oil (PO), palm kernel oil (PKO), and coconut oil (CO) were sourced from three regions in Ghana. The concentrations of 3-MCPDE and GE were quantified using the indirect method with gas chromatography-mass spectrometry. Subsequently, the statistical distribution functions of the concentrations of the esters were fitted using the Palisade @risk software. The relationships between the esters in the oils were determined using the Pearson correlation coefficient. The results showed no correlation (p > 0.05) between the concentrations of 3-MCPDE and GE. However, 18–60 % of the sampled PO contained 3-MCPDE above the European Commission's 2.5 mg/kg limit. In comparison, 24–35 % of the PO contained GE at levels exceeding the Commission's 1 mg/kg limit. Similarly, 25–35 % of PKO samples had GE concentrations above the limit. CO was the least contaminated oil, with little or no evidence of 3-MCPE and GE formation. Though the most frequently occurring (modal) concentrations of the esters were below the limits imposed by the Commission, it is the 95th percentile level of concentrations, especially for PO, that pose a health concern. Serious education and control must be exercised over the production of PO to enhance safety at the national and international markets.

## 1 Introduction

While processing vegetable oil by removing color, taste, and odor to obtain refined products, both desirable and undesirable chemical changes occur in the oil [1]. These undesirable changes include the formation of contaminants such as 3-monochloropropane-1,2-diol fatty acid esters (3-MCPDE) and glycidyl esters (GE). These contaminants are produced at high temperatures during the refining process, particularly during deodorization of the vegetable oils [2]. These undesirable contaminants are generally formed from acylglycerols. However, it is suggested that 3-MCPDE is a precursor to GE and that heat-treated oils with high 3-MCPDE may also have high GE [3]. These contaminants can also be produced in minute quantities at relatively low temperatures via a hydrolytic alternative pathway induced by lipase in the presence of water, oil, and sodium chloride [4,5]. Triacylglycerols (TAG) hydrolysis occurs during the maturation, harvesting, and transportation of oil fruits or seeds. Fruit oils are more susceptible to hydrolytic reactions than seed oils due to prolonged water exposure, resulting in notably high levels of 3-MCPDE and GE [5,6]. These contaminants are of serious public health concern due to the ability of the esters to be enzymatically converted to 3-monochloropropane-1,2-diol (3-MCPD) and 2,3-epoxy-1-propanol (glycidol) upon ingestion [7,8]. In several animal studies, glycidol is both genotoxic and carcinogenic due to its reactive epoxide group [9,10]. Studies have shown that 3-MCPD induces testicular toxicity [11], neurotoxicity [12] and nephrotoxicity [13] in rats. Both 3-MCPD and glycidol have been classified by the International Agency for Research on Cancer (IARC) as possible human carcinogen (group 2B) and probable genotoxic human carcinogen (group 2A), respectively [14]. As a result, the European Commission established respective maximum limits of 2.5 mg/kg and 1 mg/kg for 3-MCPD/3-MCPDE in combination and GE in vegetable oils [15].

In many countries, 3-MCPDE and GE have been detected in edible oils such as olive, corn, sunflower seed, peanut, and rape seed oils. Recent studies in Poland found very high concentrations of GE in edible oils, up to 44.3 mg/kg [16]. Similarly, over 2.5 mg/kg of 3-MCPDE was detected in edible oils available in Chinese markets [6,17]. Edible oils in the United States were reported to contain 3-MCPDE and GE as high as 7.2 and 10.5 mg/kg, respectively [1]. Due to the risks associated with consuming 3-MCPDE and GE, many countries have included testing of these contaminants in their routine food surveillance programs while encouraging edible oil producers to adopt safer production measures [18].

Artisanal oils are the world's most widely consumed edible oils [19]. In Ghana, they form about 80 % of the annual edible oil production [20]. These oils are highly valued in the local markets for their unique organoleptic qualities, for which they are an indispensable component of several traditional dishes [19]. However, their processing operations are characterized by poor transportation and storage conditions, unregulated processing duration and temperature, as well as the addition of taste enhancers, e.g., sodium chloride [21,22]. Perceived as a poor safety product, most of the oils produced by artisanal processors are not widely utilized in the food industry [20,23]. International organizations such as the European Commission have recently raised public health concerns about Zomi, an artisanal palm oil (PO) originating from Ghana, for containing 3-MCPDE as high as 4.45 mg/kg [24]. It was, however, unclear whether this finding was an isolated incidence or regular occurrence in Ghana's artisanal oils, which are predominantly PO, palm kernel oil (PKO) and coconut oil (CO) [25]. To fill this information gap, this study sought to investigate the occurrence and distribution of 3-MCPDE and GE in these artisanal oils to ensure consumer safety.

## 2 Materials and methods

### 2.1 Materials

#### 2.1.1 Sample collection

Artisanal oil samples were collected from 5 to January 23, 2023, intentionally timed to coincide with the peak season of oil palm production (January and May). Information on the intricacies of their production was also collected. In all, 114 artisanal vegetable oil samples, comprising 64 PO, also known as Zomi, 17 PKO, and 33 CO, were randomly purchased from two major markets in the Greater Accra, Eastern, and Volta regions of Ghana (Table 1).

**Table 1:** Sampled artisanal vegetable oils from the three regions of Ghana.

Oil type	Number of oil samples per region			Total number of samples
	Greater Accra	Eastern	Volta	
PO	22	17	25	64
PKO	9	4	4	17
CO	11	8	14	33

**Fig. 1:**
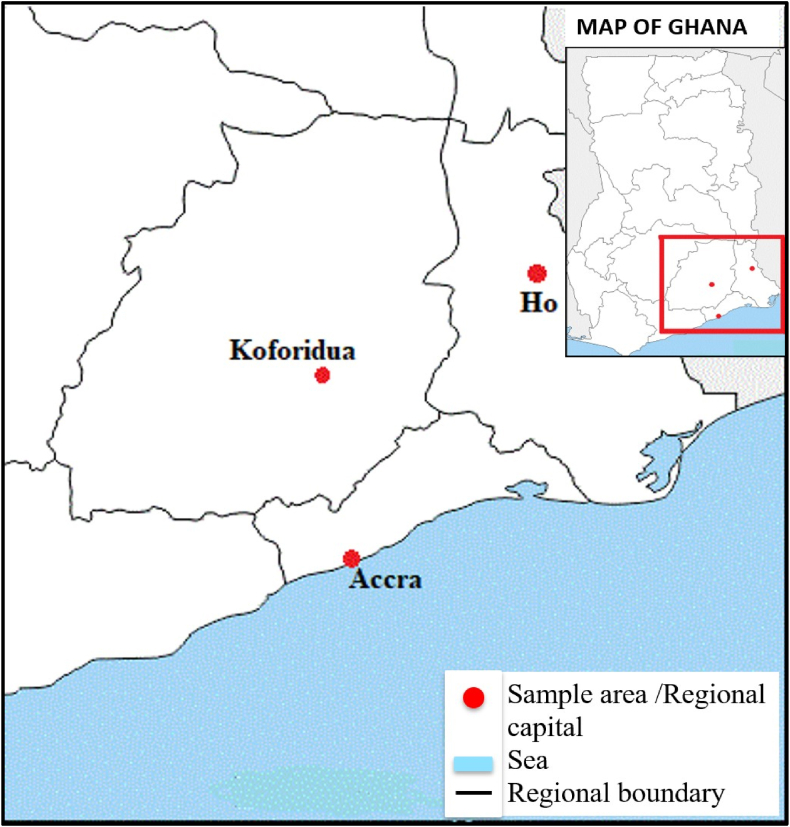
Map of Southern Ghana showing sampling locations.

Sampling was done in the capital cities of these regions, namely Accra (Greater Accra region), Koforidua (Eastern region), and Ho (Volta region) (Fig. 1). In each region, sampling was done on market days that attracted traders and producers from all surrounding towns and villages. Information gathered revolved around the following:

The consensus for the production of the PO was that the fresh palm fruit bunches were manually thrashed, stored for 3–6 days at room temperature, and cooked between 1 and 2 h. Fruits were pounded in a wooden mortar and kneaded using additional water to allow the oil to separate as an emulsion. The oil emulsion was skimmed off, and extra water was added to wash off the remaining emulsified oils on the kernels and fibers. After pooling all the washings, the emulsion was boiled to remove moisture. Sodium chloride was added based on the producer's discretion and heated further. After cooling, the PO was collected and bottled.

The PKO was generally produced after the palm nuts were sun-dried for several days. It was explained that the longer the drying period, the easier it was for the kernels to be removed mechanically from the shells. The shells and kernels were mixed in a clay bath to form a dense concentrate, which caused the kernels to float on the surface while the heavier shells sank. The floating kernels were scooped with baskets, washed thoroughly, and dried. The kernels were either fried in old oil or roasted at high temperatures in a galvanized pot, then milled into a paste, mixed with a small amount of water, and boiled to liberate the oil. The liberated oil was skimmed and allowed to cool, after which it was bottled.

In the production of CO, the mature coconuts were dehusked and cracked to remove the meat from the shell, usually using a cutlass. The meat was crushed using a mill or a grater, after which water was added and kneaded to obtain the milk. The milk was drained through a sieve and kept for 2–3 days to ferment, after which it was warmed to expel the melted oil, which was skimmed off and heated further to remove moisture, cooled and bottled.

#### 2.1.2 Reagents

Analytical chemical reagents such as anhydrous sodium bromide (NaBr), anhydrous tetrahydrofuran (THF), n-heptane and 2,2,4-trimethylpentane were purchased from Surechem Products Ltd (Suffolk, England). Phenylboronic acid (PBA), methanol, sodium hydrogen carbonate (NaHCO_3_), anhydrous sodium sulphate, acetone and sulphuric acid were also purchased from Sigma-Aldrich Co. (Missouri, USA). Toluene and sulphuric acid (H_2_SO_4_; 95 %) were purchased from Sigma-Aldrich (Steinheim, Germany). Analytical standards 1,3-dipalmitoyl-2-chloropropanediol (3-MCPDE), glycidyl palmitate (GE), isotopically labelled 1,3-dipalmitoyl-2-chloropropanediol-d_5_ (3-MCPDE-d_5_) and glycidyl palmitate-d_5_ (GE-d_5_) were purchased from Toronto Research Chemicals Inc. (Toronto, Canada).

## 3 Methods

### 3.1 Sample preparation

Sample preparation and the working solutions of 3-MCPDE and GE determination were performed based on the American Oil Chemists Society (AOCS) standard indirect method Cd 29a-13 as described by Ref. [26]. Oil samples (100 mg) were weighed into glass tubes and spiked with 100 μL of mixed internal standard solution (3-MCPDE-d_5,_ 2.5 μg/mL and GE-d_5,_ 2.5 μg/mL). The mixture was dissolved in 2 mL of THF. A volume of 30 μL of an acidified aqueous solution of NaBr (3.3 mg/mL, 5 % ^v^/_v_ H_2_SO_4_) was added to the mixture and incubated at 50 °C for 15 min to convert the GEs to 3-monobromopropanediol esters (MBPDEs). The reaction was stopped by adding 3 mL of 0.6 % ^v^/_v_ NaHCO_3_, and then 2 mL of n-heptane was added to extract the compound of interest. A nitrogen (N_2_) stream at 40 °C evaporated the extract to dryness, and the resulting residue was dissolved in 1 mL THF. Consequently, a transesterification reaction was performed by adding 1.8 mL of methanolic sulphuric acid (1.8 % v/v) to the solution and incubating at 40 °C for 16 h to obtain 3-MCPD and glycidol in their free forms. The reaction was stopped using 0.5 mL of NaHCO_3_ (9 %), and the organic solvents were evaporated using an N_2_ stream at 40 °C. Liquid-liquid extraction (2 mL 20 % ^v^/_v_ Na_2_SO_4_; 2 × 2 mL n-heptane) was used to separate the fatty acid methyl esters from the sample. Derivatization was done at room temperature by adding 200 μL of PBA solution (250 mg/mL acetone; H_2_O 19:1^v^/_v_) in an ultrasonic bath for 5 min. n-heptane (2 × 1 mL) was used to extract the phenylboronate derivatives and afterwards evaporated at 40 °C under a stream of N_2_. The sample was finally re-dissolved in 300 mL of 2,2,4-trimethylpentane. The sample (1 μL) was then injected into the GC-MS.

### 3.2 Instrumentations

GC-MS analyses were performed on a GC2010 Plus gas chromatograph coupled to a QP2020 mass spectrometer (Shimadzu, Kyoto, Japan). An aliquot of 1 μL of the sample extract was injected at 260 °C in splitless mode. A capillary column VF-5MS 30 m × 0.25 mm (0.25 μm) (Agilent Technologies) was used for the separation. The carrier gas used was helium at a flow rate of 1.2 mL/min while the oven's temperature was programmed as 60 °C (kept for 1 min), 6 °C/min to 150 °C (kept for 2 min), 30 °C/min to 300 °C (kept for 10 min). Detection was performed using selected ion monitoring (SIM) following positive electron impact ionization (70 eV). The ions monitored for quantitative analysis were: *m*/*z* 147, 196 and 198 for derivatized 3-MCPD; *m*/*z* 150, 201 and 203 for derivatized 3-MCPD-d_5_; *m*/*z* 147 and 240 for derivatized 3-MBPD; *m*/*z* 150 and 245 for derivatized 3-MBPD-d_5_.

### 3.3 Method validation

The method was validated for linearity, repeatability, recovery, the limit of quantification (LOQ) and the limit of detection (LOD). For 3-MCPDE and GE, linearity was assessed in the 0.05–4.0 mg/kg range and 0.03–6.0 mg/kg, respectively (six calibration levels). The LOQ and LOD were estimated as three- and six-fold standard deviations of six blank extra virgin olive oils, each measured once. To assess the repeatability and recovery, the blank sample was spiked with 3-MCPDE and GE at concentrations of 0.1, 0.5, 2.0, and 0.2, 1.0 and 5.0 mg/kg, respectively (six replicates for each concentration level) and analyzed. The repeatability was expressed as a relative standard deviation.

### 3.4 Data analysis

Samples with 3-MCPDE and GE concentrations below the LOD were assumed to be 0.5 × LOD [27]. Sample sizes of PKO obtained from Eastern (n = 4) and Volta (n = 4) regions were inadequate for valid individual probabilistic estimation. Hence, their data were combined. The concentration data of the 3-MCPDE and GE were fitted using Palisade @Risk 8.0 for Excel (Palisade Corporation, Raleigh, NC, USA) to obtain the statistical distribution functions from which the minimum, maximum, mean, mode, 5th percentile and 95th percentile were studied. The Pearson correlation coefficient was used to determine the relationship between 3-MCPDE and GE content in oils. A p-value <0.05 was considered statistically significant.

## 4 Results and discussion

### 4.1 Method validation

The results from the method validation show linearity for the selected concentrations with correlation coefficients 0.9998 for 3-MCPDE and 0.9995 for GE. The LOD and LOQ numerical values were 0.02 and 0.05 mg/kg for 3-MCPDE and 0.02 and 0.03 mg/kg for GE, respectively. A summary of the results obtained from the validation is shown in Table 2.

**Table 2:** Validation data of the analytes: 3-MCPDE and GE of the sampled vegetable oils.

Analyte	Linear range (mg/kg)	Correlation coefficient (r^2^)	LOD (mg/kg)	LOQ (mg/kg)	Repeatability (%)	Recovery (%)
3-MCPDE	0.05-4.0	0.9998	0.02	0.05	6.1	113.9
GE	0.03–6.0	0.9995	0.02	0.03	8.4	91.7

### 4.2 Correlation between 3-MCPDE and GE

Fig. 2a, Fig. 2b, Fig. 2c(a–c) are the scatter plots showing the correlations between the concentrations of the

**Fig. 2a:**
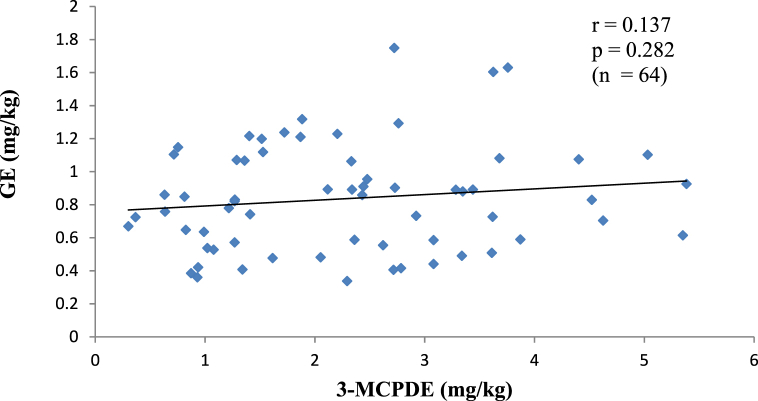
A plot of the amount of 3-MCPDE and GE in PO.

**Fig. 2b:**
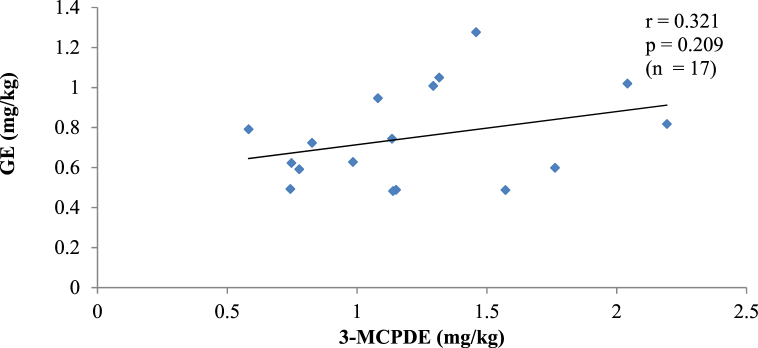
A plot of the amount of 3-MCPDE and GE in PKO.

**Fig. 2c:**
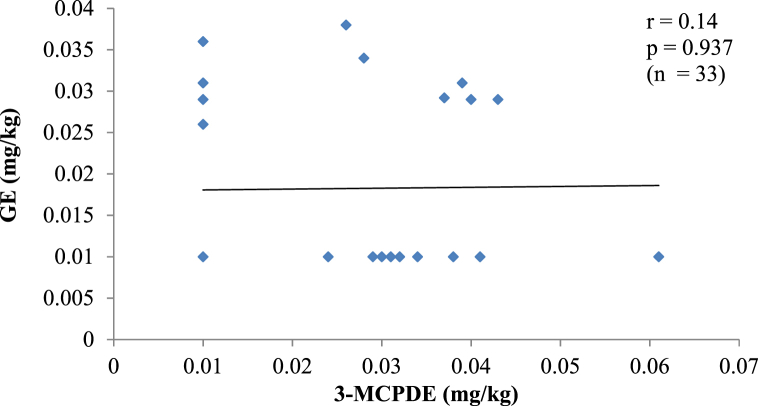
A plot of the amount of 3-MCPDE and GE in CO.

Two processing contaminants in PO, PKO and CO. The correlation between 3-MCPDE and GE concentrations in each oil type was investigated since 3-MCPDE is believed to be a precursor of GE [3]. The results show no correlation (p > 0.05) between the two contaminants in all the oils. Similar findings in instant noodles, fried chicken and confectionery have also been made, as well as in marine oils [3,28]. A correlation between 3-MCPDE and GE would suggest that 3-MCPDE formation also influences the GE formation in the oils. However, unlike 3-MCPDE, GE is mainly formed from diacylglycerols (DAG) without the involvement of chlorine ions [29]. In addition, GE formation starts at about 200 °C. It rises exponentially at temperatures above 230 °C, whereas 3-MCPDE formation occurs at a lower temperature (160–200 °C), and the formation rate does not increase as the temperature rises [3]. Given the distinct mechanisms for 3-MCPDE and GE formation, there is unlikely a direct precursory relationship between the contaminants.

### 4.3 Occurrence of 3-MCPDE and GE

It has been well established that 3-MCPDE and GE are produced in edible oils during deodorization. Usually, unrefined oils contain low amounts of these compounds, which most analytical methods would not detect [1,30]. This study investigated 3-MCPDE and GE levels in artisanal PO, PKO and CO, whose production methods do not involve deodorization. Table 3 shows the results obtained for sampled oils from the Accra, Koforidua, and Ho markets. Both 3-MCPDE and GE of the sampled oils presented varying statistical distribution functions, predominantly Pareto, though other functions were observed for some markets. The concentrations of the contaminants varied widely from one sample to another, ranging from below LOD (<0.02) to 5.39 mg/kg for 3-MCPDE and <0.02–1.75 mg/kg for GE.

**Table 3:** Levels (mg/kg) of 3-MCPDE and GE in oils obtained from three regions in Ghana.

Types of hazards	Types of oils	Statistical functions	Min	Max	Mean	Mode	Median	5th P^a^	95th P^a^
**Accra market only**									
	PO	Triang (0.301,0.301,5.1876)	0.30	4.63	2.04	2.33	1.88	0.37	3.68
**3-MCPDE**	PKO	Pareto (2.1334,0.743)	0.74	2.19	1.27	0.78	1.29	0.74	2.19
	CO	Pareto (1.6058,0.01)	0.01	0.04	0.02	0.01	0.02	0.01	0.04
**GE**	PO	Triang (0.338,0.338,1.7762)	0.34	1.60	0.85	0.88	0.76	0.36	1.32
	PKO	Pareto (2.2769,0.493)	0.49	1.28	0.80	0.59	0.74	0.49	1.28
	CO	Pareto (2.4816,0.010)	0.01	0.04	0.02	0.01	0.01	0.01	0.04
**Koforidua market only**									
**3-MCPDE**	PO	Pareto (1.608,0.754)	0.75	3.62	1.59	1.26	1.27	0.75	3.62
	CO	Pareto (1.2140,0.010)	0.01	0.06	0.03	0.01	0.03	0.01	0.06
**GE**	PO	Uniform (0.3352,1.2498)	0.39	1.20	0.79	0.40	0.78	0.39	1.20
	CO	Pareto (2.3046,0.010)	0.01	0.04	0.02	0.01	0.01	0.01	0.04
**Koforidua and Ho markets only**									
**3-MCPDE**	PKO	Ext Value (0.97768,0.33281)	0.58	2.04	1.17	1.13	1.08	0.58	2.04
**GE**		Uniform (0.40629,1.09671)	0.48	1.02	0.72	0.50	0.69	0.48	1.02
**Ho market only**									
**3-MCPDE**	PO	Uniform (0.52042,5.5796)	0.72	5.39	2.97	2.36	2.73	0.81	5.35
	CO	Pareto (1.7164,0.010)	0.01	0.04	0.02	0.01	0.01	0.01	0.04
**GE**	PO	Triang (0.4080,0.4080,1.8755)	0.41	1.75	0.85	0.91	0.86	0.44	1.63
	CO	Pareto (2.1090,0.010)	0.01	0.03	0.02	0.01	0.01	0.01	0.03

*The maximum value for 3 MCPDE and GE is 2.*5 mg/kg *and* 1 mg/kg*, respectively, according to European Commission* [15].

a Percentile.

This wide variation in the levels of contaminants from various sources is possibly due to the processing diversity among artisanal oil producers [19]. Generally, the levels found in this study are higher than the 3-MCPDE (<LOD – 0.09 mg/kg) and GE (<LOD – 0.03 mg/kg) levels reported for unrefined vegetable oils [1]. The contamination levels are typically similar to those found in refined vegetable oils and fats such as palm oil, soyabean oil, maize oil, camellia and margarine [6,31]. Some studies, however, found higher levels in refined palm oils. For instance, 7.23 mg/kg and 10.52 mg/kg of 3-MCPDE and GE were reported in palm oil [1], whereas GE was 4.31 mg/kg in similar oil [32]. The European Commission has demarcated maximum permissible levels of 3-MCDPE and GE in vegetable oils as 2.5 mg/kg and 1 mg/kg, respectively [15]. In this study, the most frequently occurring (modal) concentration of 3-MCPDE and GE varied from <0.02 to 2.36 mg/kg and <0.02–0.091 mg/kg, respectively, for the oils from all the markets. Thus, under the Commission's guidelines, most of the three artisanal oils available in the markets contained 3-MCPDE and GE within the regulatory limits. The mean 3-MCPDE and GE contaminations in oils were in the order PO > PKO > CO, with CO being the least contaminated oil, with little or no evidence of 3-MCPDE (<0.02–0.06 mg/kg) and GE (<0.02–0.04 mg/kg) formation. This was expected since artisanal CO production requires less heating than PO and PKO production. PO collected from the Ho markets had the highest mean 3-MCPDE content of 2.97 mg/kg, followed by PO from Accra markets with a mean 3-MCPDE content of 2.04 mg/kg. Also, the 95th percentile concentration of 3-MCPDE and GE in all PO samples were above the European Commission's thresholds, with the highest concentrations (3-MCPDE: 5.35 mg/kg; GE: 1.63 mg/kg) detected in PO samples from Ho markets. The peculiar contamination of PO samples from Ho markets may be because PO is a staple oil in the Volta region, and its usual production method is unique to the region [33]. In the case of PKO, all samples showed a 95th percentile 3-MCPDE concentration below 2.5 mg/kg, whereas the 95th percentile GE concentrations were above 1 mg/kg. Implicitly, PO and PKO containing 95th percentile concentrations of 3-MCPDE and GE marketed and used directly as food ingredients in Ghana and across West Africa pose a great threat to consumer health. The situation is even more precarious when such vegetable oils find application in infant foods where stricter regulation is imposed [34]. Compared to Malaysia, the artisanal vegetable oils produced in the study area are hardly regulated. Unlike Ghana, Malaysia set a timeline to comply with the maximum regulatory limits that came into force in 2020. Their regulations set the upper limit for 3-MPCDE in PO, PKO, and GEs in PKO to 2.5, 1.25, and 1 mg/kg [35].

Generally, the study found PO to be the most contaminated oil and agrees with previous studies, which identified refined palm oil as the most contaminated [1,36]. The maximum 3-MCPDE concentration in the PO was relatively higher than the 4.45 mg/kg found by the European Commission in artisanal PO originating from Ghana [24]. Notably, 18–60 % of the PO samples contained 3-MCPDE at concentrations exceeding the Commission's threshold. Similarly, 24–35 % of the PO had GE concentrations exceeding the threshold recommended by the Commission. The contamination in the PO samples is likely due to their production materials. Palm fruit pulp, used to produce PO, contains abundant water and active lipases. These lipases promote the hydrolysis of TAG to diacylglycerols (DAG) and monoacylglycerols (MAG), which are precursors for forming 3-MCPDE and GE [6]. The DAG, for instance, makes up between 6 and 10 % of palm oil, which is higher than the DAG content of other vegetable oils, typically between 1 and 3 % [37]. The hydrolysis of TAG is facilitated by more extended palm fruit storage, a common practice among farmers and processors in Ghana [20]. Additionally, producing artisanal PO involves using sodium chloride as a taste enhancer, which is reported to accelerate the rate of 3-MCPDE formation in food. The salt facilitates the detachment of ester bonds from acylglycerols during heating and provides chlorine ion that reacts with the glycerol backbone to form 3-MCPDE [37].

As observed in PO, a substantial percentage of PKO samples (25–35 %) across the three regions had GE concentrations above the Commission's threshold. The GE and 3-MCPDE occurrence in these samples may be due to unregulated processing temperature and chlorinated substances in the starting palm kernels [17,38]. The chlorinated substances in palm fruits may arise from endogenous production during maturation [29] or external sources like fertilizer and pesticide application on the farm [19,39]. Notwithstanding, the exact factors influencing 3-MPDE and GE formation in the oil must be investigated. In Ghana, however, the ingestion of PKO in food has drastically declined over the years, with the product finding increasing uses in the lamp oil and soap industry [40]. This trend explains the scarcity of PKO samples from the markets.

## 5 Conclusion

The study found 3-MCPDE in some artisanal PO at levels comparable to the level reported by the European Commission. While the frequently occurring (modal) concentrations of 3-MCPDE and GE in the three artisanal oils were within the regulatory limits, significant quantities of PO and PKO, particularly those sampled from the Volta and Greater Accra regions, were dangerously high. Conversely, artisanal CO presented a negligible amount of these contaminants. These findings suggest potential health risks for consumers of Ghanaian artisanal oils, particularly PO. Further investigation is needed to identify the factors influencing 3-MCPDE and GE formation in artisanal PO and PKO, which would inform the development of appropriate mitigation against their occurrence. This would improve the safety of the oils and help producers compete in the market based on high-quality products. The study also recommends public education programs for producers and consumers on the health risks associated with 3-MCPDE and GE. Additionally, regulations should be implemented to ensure the safety of artisanal oils in Ghana.

## Data availability statement

Data will be made available on request.

## CRediT authorship contribution statement

**Daniel Sitsofe Yabani:** Writing – original draft, Resources, Project administration, Methodology, Investigation, Formal analysis, Data curation, Conceptualization. **Isaac Williams Ofosu:** Writing – review & editing, Supervision. **Gloria Mathanda Ankar-Brewoo:** Writing – review & editing, Supervision. **Herman Erick Lutterodt:** Writing – review & editing, Supervision.

## Declaration of competing interest

The authors declare that they have no known competing financial interests or personal relationships that could have appeared to influence the work reported in this paper.
